# Phenotypic characterization of idiopathic epilepsy and epilepsy of unknown cause in Irish Setters

**DOI:** 10.3389/fvets.2022.1066094

**Published:** 2022-12-12

**Authors:** Marta Plonek, Montse M. Diaz-Espineira, Quirine E. M. Stassen, Koen M. Santifort, Peter A. J. Leegwater, Paul J. J. Mandigers

**Affiliations:** ^1^Neurology Section, Evidensia Hospital Arnhem, Arnhem, Netherlands; ^2^Department of Clinical Sciences, Faculty of Veterinary Medicine, Utrecht University, Utrecht, Netherlands

**Keywords:** canine epilepsy, dog, idiopathic epilepsy, epileptic seizure, hereditary

## Abstract

Canine epileptic seizures are common neurological symptom presenting to veterinary practice. Idiopathic epilepsy (IE) with a suspected genetic background has been reported in several dog breeds. Although it has been reported in the Irish Setter (IS), the phenotypic characteristics have not yet been described. The aim of this study was to characterize the phenotype of IE in this breed and to trace its mode of inheritance.

Owners of IS were requested to fill in a questionnaire *via* the Dutch Irish Setter Club concerning the epileptic seizures in their dogs. The data was assessed retrospectively using descriptive statistics. Forty-eight privately owned IS dogs fulfilling tier I criteria for IE according to the International Veterinary Epilepsy Task Force of both sexes were included in the study. The mean age of seizure onset was 41 months. Five of the dogs included in the study had an onset of seizures >6 years of age. These dogs were classified with epilepsy of unknown cause (EUC). Primary generalized tonic-clonic seizures were the most common type of seizure and were seen in almost all dogs. Cluster seizures were reported in 54% of the studied population. Most owners reported pre- (56%) and post-ictal (97%) signs in their dogs. A pedigree analysis of one subpopulation was performed and traced the lineage of 13 affected IS. A segregation analysis of this population rejected a simple autosomal recessive inheritance pattern. The present study supports the occurrence of IE and EUC in the IS. The results provide clinical insight into epileptic seizures in this breed and may be a starting point for further, including genetic, analysis.

## Introduction

Canine epilepsy is a common neurological diseases encountered in veterinary practice. The International Veterinary Epilepsy Task Force (IVETF) has defined epileptic seizures as manifestation(s) of excessive synchronous, usually self-limiting epileptic activity of neurons in the brain (1) and epilepsy as a disease of the brain characterized by an enduring predisposition to generate seizures. Epilepsy in dogs can be classified as structural, caused by an underlying intra- or extra-cranial pathology or idiopathic epilepsy (IE). The latter serves as an umbrella term encompassing genetic epilepsy (where a causative gene for epilepsy has been identified), suspected genetic epilepsy (where a genetic influence is suspected but not confirmed) and epilepsy of an unknown cause (EUC; age at seizure onset < 6 months old or >6 years old and where no known cause for epilepsy has been identified) (2).

Canine IE has a prevalence ranging between 0.5 and 5% in most canine populations (3, 4). Canine genetic epilepsy with an autosomal recessive inheritance pattern has been confirmed in the Lagotto Romagnolo and Rhodesian Ridgeback (5, 6). To the authors' knowledge, only a few studies have been published in which risk loci for IE have been described. A common risk haplotype in the *ADAM 23* gene has been reported for the Belgian Shepherds, the Schipperke, Finnish Spitz, Beagle, Australian Shepherd, Kromfohrländer, Labrador Retriever, and Whippet (7, 8).

Two recent studies identified additional risk haplotypes in the Belgian Shepherd: a significant association between canine IE and a risk haplotype on chromosome 14 (9, 10).

If a genetic cause or risk factor of epileptic seizure (ES) is lacking, patterns and characteristics in specific canine breeds, such as the age of disease onset as well as prevalence of status epilepticus (SE) or cluster seizures (CS), may induce genetic research into more targeted treatment and clear breeding guidelines.

The Irish red and white Setters are a native Irish breed considered highly vulnerable due to its limited population (11). The Dutch Irish Setter Club was registered in 1915. The breed became increasingly popular in the Netherlands in 1930s and experienced a boom in the 1970s with a high demand for pups, leading to as many as 1,700 new pups being born per year. That number decreased in the 1980s to 300–400 new pups per year and a steady decline in the litter numbers has been observed until today (with an average annual birth rate at 150–200 puppies). Many of the dogs were not bred according to the recommendations of the Dutch Irish Setter Club.

To date, IE has been reported to occur in the IS (8, 12–14) but its phenotypic characteristics have not been previously described. Therefore, the aim of this study was to characterize IE in this breed and to determine the mode of inheritance.

## Materials and methods

A cross-sectional analysis of idiopathic epilepsy in the Dutch population of IS was performed, including analysis of the occurrence of seizures, clinical outcome, and family relations. Cases were collected *via* the Dutch Irish Setter Club, who were asked to send out a questionnaire to owners of living and deceased IS dogs with a history of epileptic seizures.

### Recruitment of cases and inclusion criteria

The questionnaires were filled out on paper or online in the years 2002–2021. All data was assessed retrospectively. Dogs were included in the study if they fulfilled a tier I confidence level for the diagnosis of IE, or the equivalent thereof (15). Dogs with an onset of seizures above 6 years of age were required to fulfill tier I criteria and undergo brain magnetic resonance imaging, ruling out structural causes for the seizures, whereby they were classified with epilepsy of unknown cause (EUC). Furthermore, all dogs were required to have traceable pedigree information, no history or current treatment for other neurological disorders, and a filled-out owner questionnaire. All the dogs underwent a clinical and neurological examination after seizure onset, which was performed either by the primary caretaker or one of the authors (MP, MDE, QS, KS, PM) during referral, and was deemed normal. A blood examination was performed in all recruited cases and included a complete blood count, basic biochemistry including liver function tests and electrolyte measurements. Next to this, owners were asked to provide a MOV or MP4 recording of their dog's seizure or if this was not available to confirm the seizure type by looking at pre-recorded generalized tonic-clonic seizure and/or focal seizures of Irish Setter's. All recordings made by the owners were examined by a board-certified diplomate of the European College of Veterinary Neurology (ECVN) (PM). All the dogs were privately owned, and the owners gave informed consent to including their dogs in the study.

The questionnaire gathered the following data: pedigree information, date of birth, sex, neuter status, body weight of the dogs, owner and primary veterinarian information, epileptic seizure presentation: the appearance of pre- and post-ictal signs, the frequency, duration and time of seizures, the time of day they occurred, the longest seizure episodes, any correlation with feeding or other possible triggers, treatment, and other medical conditions. The questionnaire included closed multiple choice and dichotomous (yes/no) questions as well as several open questions. Dogs were excluded from the study if they did not meet the previously listed inclusion criteria.

Data from the questionnaires was assessed independently by an ECVN veterinary neurology resident (MP) and board-certified diplomate of the ECVN (PM).

Pedigree analysis was performed on a subpopulation where common ancestors could be traced back several generations. Dogs included in this study were marked as “affected,” while their littermates were marked as “not reported as affected.”

### Statistical analysis

Data were analyzed using descriptive statistics using Microsoft Office Excel^®^ 2021 version 16.55, Microsoft, the Netherlands. Data distribution was analyzed using the Shapiro-Wilk test. Data were presented as means with standard deviation (SD) or medians (with ranges). Student's *t*-test was used to compare normally distributed data, whereas non-normally distributed variables was assessed using the Mann-Whitney U test. Statistical significance was determined at *p* < 0.05.

## Results

### Study population

**Figure 1:**
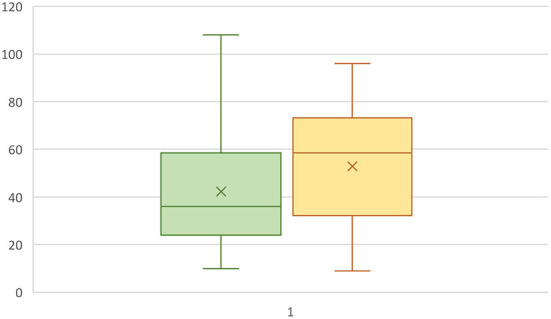
Box plot representing the age at seizure onset in male **(green)** and female **(yellow)** IS dogs. The Y-axis represents months.

Fifty-eight questionnaires from IS owners were filled out. The questionnaire responses were fully analyzed to confirm their eligibility for inclusion in the study. Of the respondents, eight were excluded for not meeting the inclusion criteria and two were excluded for reporting signs other than those consistent with IE (fly-catching, behavioral disorders), leaving forty-eight dogs included in the study. Twenty-one of the dogs were females (44%), 15 of which were not spayed (71%). Twenty-seven dogs were male (56%), 19 of which were intact (70%). Seizure onset was between 1 and 6 years in 36 dogs (75%), ≤ 1 year in four dogs (8%) and ≥6 years in seven dogs (14.5%). The mean age of seizure onset ±SD was 41 ± 24 months (range 9–108 months) without a statistically significant difference in the age of onset between males and females (*P* = 0.28) or between intact or neutered dogs (*P* = 0.48), Figure 1. There were also no statistically significant differences in the age of seizure onset between intact vs. neutered males and females (p=0.11 for males, p=0.65 for females). The mean body weight of the included IS was 31.0 ± 4.2 kg (mean ± SD).

### Owner observations

Twenty-four owners had made a film of their dogs' seizures. All others confirmed it using the provided example films. Twenty-six (54%) IS were reported to have cluster seizures (CS) and two of the dogs (4%) were reported to have status epilepticus (SE) (Table 1). One dog (2%) was reported to have focal motor seizures (facial muscular twitching). The ES were reported to last up to 5 min in most of the dogs (*n* = 36, 75 %), while they lasted up to 15 min in the remaining dogs (*n* = 12, 25%). They occurred out of rest in 28 dogs (62%). The seizures were reported to occur mostly at night in 19 dogs (40%), followed by the morning and evening (*n* = 14 each, 29%). The seizure frequency varied from one seizure per 3 days to one per year and was not specified by some owners. The median seizure interval was 3 weeks in those patients, where a regular seizure interval was reported (*n* = 21, median 21 days, range 3–365 days).

**Table 1:** The occurrence of CS as reported for male and female IS.

	**Number of dogs (*n*)**	**%**
		**100%**
Reported cluster seizures	26	54%
Females	12	46%
Entire	9	75%
Sterilized	3	25%
Males	14	53%
Intact	11	79%
Neutered	3	21%

In those dogs where an aura could be observed (*n* = 27, 56%) the owners reported signs including restlessness, being overly affectionate (*n* = 7, 26%), an absent look, panting and seeking a quiet spot. This lasted for a median of 10 min (range 0.2–48 min). A detailed list of the reported signs is presented in Table 2.

**Table 2:** Most common signs seen during the generalized seizure.

	**Number of dogs (*n*)**	**%**
	**48**	**100%**
Falling down, stiff, outstretched limbs, leg cramps	48	100%
Paddling	24	50%
Urination	34	71%
Defecation	7	15%
Foaming from the mouth	33	69%
Twisting of the neck	12	25%
Face muscle cramps/twitches	20	42%

Primary generalization was reported in all but one dog (*n* = 47, 98%) and included tonic-clonic and autonomic (urination, defecation, foaming) signs. Both signs were seen in most of the dogs (*n* = 46, 96%), the autonomic signs were not seen in two dogs (4%) (see Table 3 for the most common presentation). The median ictus duration was 4 min (range 1–15 min). A post-ictal phase was seen in 43 dogs (90%) and featured weariness, restlessness, disorientation, thirst, or hunger and seeking the owner (Table 3). This was reported to last for 2–120 min.

**Table 3:** Reported pre- and post-ictal signs in the studied IS population.

	**Number of dogs (*n*)**	**%**
	**48**	**100%**
**Reported pre-ictal signs**	27	56%
Seeking the owner	7	17%
Restlessness	8	30%
Seeking a quiet spot	3	8%
Autonomic (vomiting, hypersalivation)	2	7%
**Reported post-ictal signs**	43	90%
Disorientation	36	75%
Thirst or hunger	15	31%
Restlessness	13	36%
Weariness	23	48%
Seeking owner	19	40%
Other (aggression, blindness)	3	8%

Owners were also asked to assess awareness of their dogs during the ES. Thirty-two (67%) of the owners thought their dogs were not conscious, some owners thought their dogs were conscious (*n* = 8, 17%) and an equal number of owners were not sure of their dog's level of consciousness (*n* = 8, 17%). No relation between the time of feeding and seizure occurrence was found.

The owners reported an unchanged seizure duration with time in 24 dogs (50%), a decreased seizure duration with time in seven dogs (*n* = 7, 15%) and an increased seizure duration with time in four dogs (8%). Data regarding changes in seizure duration was unavailable for 13 dogs (27%). The seizure frequency decreased with time in 18 dogs (38%, 14 of which (78%) were treated medically), increased with time in 15 dogs (31%) (11 of which (73%) were treated medically) and remained unchanged in seven dogs [15%, four of which were treated medically (57%)]. Data regarding seizure frequency was unavailable for eight dogs.

### ASM treatment

ASM treatment was administered in the majority of the cases (*n* = 34, 71%) and consisted of phenobarbital (*n* = 26, 54%), imepitoin (*n* = 9, 19%), potassium bromide (*n* = 8, 17%) and levetiracetam (*n* = 1, 2%). Thirteen of the dogs (27%) were not treated medically, two of which were reported to have CS. Combination therapy was reported in seven dogs (*n* = 15%). Seizure treatment was not the focus of the study; hence the exact ASM dosages, treatment regimens and outcomes were not analyzed further. Spontaneous remission of the seizures was reported in two dogs (4%). One of these dogs was reported to have had CS.

### Pedigree analysis

**Figure 2:**
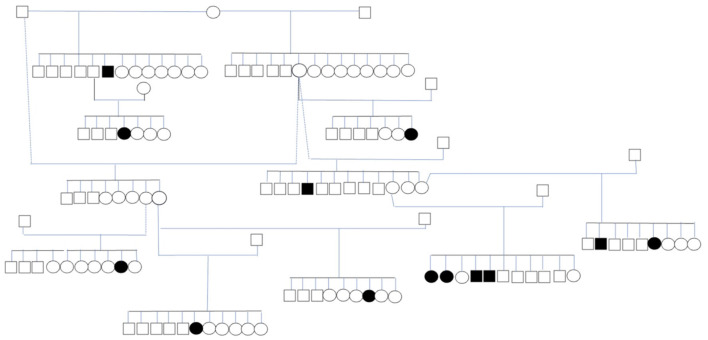
Subpopulation of IS with 13 affected dogs. The circles represent females, the squares represent males, shapes filled in black represent dogs with ES.

Thirteen affected dogs from 11 litters were closely related (Figure 2). The dogs with IE in the pedigree were related through at least a parent or grandparent to other dogs with IE. The dogs included in the pedigree analysis included eight females and five males. The mean age at seizure onset ±SD in this subpopulation was 28 ± 20 months (range 9–58 months). The pedigree indicates that inherited factors play an important role in the etiology of the disease. However, the proportion of affected dogs in these litters suggest that the inheritance is not simply monogenic (Table 4).

**Table 4:** Segregation analysis of the studied affected littermates.

**Litter**	**Affected siblings**	**Unaffected siblings**	**% Affected in litter**
1	1	12	7.6%
2	0	14	0%
3	1	6	14.2%
4	1	6	14.2%
5	0	8	0%
6	1	11	8.3%
7	1	9	10%
8	1	10	9.1%
9	1	8	11.1%
10	4	7	36.3%
11	2	7	22.2%
Total affected	13	98	11.7%

## Discussion

This is the first study providing a phenotypic analysis of IE and EUC and its inheritance in the ISs. In the reported group of IS dogs, the mean age of seizure onset was 41 months, which is higher than reported in most reported breeds, including the Border collie (median age of onset 27 months), Vizsla (median age of onset 36 months) and Great Swiss Mountain dog (mean age of onset 28.8 months) but lower than that reported in the Standard Poodle (median age of onset 43 months) (16–19). Early-onset seizures, occurring at < 1 year, were observed in four dogs. Seven dogs with late-onset seizures occurring at or above 6 years of age, were also included in the study. Seizure onset occurring at the higher end of the range for IE (≥60 months) has been reported in other breeds of dog, including the poodle, English Springer Spaniel, Labrador Retriever, Italian Spinone, Finnish Spitz and Border Terrier (20). In a study assessing the cause of seizures in dogs above 5 years of age, primary epilepsy was diagnosed in 35% of the population (21). In another study assessing aged dogs with seizures, 29% of the dogs between 7 and 10 years old were diagnosed with cryptogenic epilepsy (22). According to the consensus of the IVETF, epilepsy in dogs with a seizure onset above 6 years of age cannot be termed IE. However, the consensus also states that IE is chiefly a diagnosis of exclusion, where other causes of seizures have been excluded. We believe IE needs to be considered in dogs with a later onset of seizures (above 6 years of age) where a genetic background is suspected. This type of epilepsy complicates selection for breeding as many cases are used for mating before the occurrence of their first epileptic seizure.

We reported a higher occurrence of IE in males than females (56 vs. 44%), which is consistent with observations in other breeds (61.6 vs. 38.4% in the Irish Wolfhound, a ratio of 1.4:1 for all epilepsy-related insurance claims in Sweden, 57–43% in the Border Collie, 1.69:1 for dogs with epilepsy of unknown origin in the UK) (3, 4, 18, 23). The occurrence of ES in IE males was not statistically significant. The majority of the dogs in this study were intact. Previous studies have suggested that neutering may positively affect seizure frequency and severity (24, 25), although one study found this not to be true for females (26). The effect of neutering on seizure frequency was not analyzed in this study.

More than half of the dogs in this study were reported to have CS (54%). This occurrence of CS was lower than that reported in the Border Collie [94% occurrence of CS in a population of 49 dogs (27), and 59% reported in another study of 114 Border Collies (18)] as well as the Dalmatian [63.6% CS (20)]. However, it was higher than in the Australian Shepherd (20% of CS) and Belgian Shepherd (33%) (28, 29). The high occurrence of CS and low occurrence of SE in the studied population is consistent with a similar IE analysis on a large population of dogs of various breeds (30). In that study of 407 dogs of five breeds, 41% of the population experienced CS and only 2.5% were reported with SE. It has been previously reported that a high occurrence of CS leads to poor outcome and euthanasia (30). Hence, the occurrence of CS warrants early treatment.

The majority of the dogs in this study were treated medically. Most of the reported seizures in the studied IS population occurred out of rest and at night, which is consistent with similar findings in other breeds (16, 20). No known triggers were reported and no association between feeding and ES were found. The latter have been reported in a group of dogs, nine of which were diagnosed with IE (31). Most (67%) of the dog owners thought their dogs were not conscious during the seizures. The assessment of consciousness during a seizure is often difficult for owners and thereby subjective. It has been suggested by the IVETF that the assessment of consciousness is not a meaningful factor in the classification of canine seizures (2).

Spontaneous seizure-freedom was reported in two dogs. One of those dogs had been reported to previously have had cluster seizures. Although uncommon, spontaneous seizure-freedom has been reported in other breeds of dogs, including the Border Collie (27). Seizure remission on treatment was reported in three dogs (6%) in that study.

Recent literature suggests that a majority of owners of epileptic dogs are able to predict an upcoming ES (32). Prodromal signs were observed by most owners in this study. The behavioral signs prior to or following a seizure are consistent with those previously reported in dogs and humans including disorientation, autonomic signs, restlessness and lethargy (33). According to the IVETF, some pre-ictal signs such as restlessness, “clinging” to the owner or anxiousness may, in truth, be behavioral focal epileptic seizures (2, 20). Advanced diagnostic techniques, such as electroencephalography, may assist in determining the nature of the behavioral changes preceding a generalized epileptic seizure, but exceeded the scope of the present study.

All dogs reported in this study had generalized seizures, which were either primary or a secondary generalization of focal seizures (in one dog). Recent analyses suggest focal seizures may pose a diagnostic challenge, as they may mimic other disorders (34). A higher occurrence of generalized seizures than focal seizures has been reported in other breeds of dog, such as the Golden Retriever, Labrador Retriever, Irish Wolfhounds and the Shetland Sheepdog (23, 35–37).

The pedigree analysis of one subpopulation of IS allowed us to trace the inheritance pattern of thirteen dogs. Most canine IE data is based on owner observations. This data provides crucial insights into epilepsy in predisposed breeds and facilitates seizure categorization. Hence, it is essential to promote owner-veterinarian collaboration to identify, treat and limit canine epilepsy and to improve the quality of life of epileptic patients. Large scale studies on canine breeds may facilitate the detection of the genetic background of IE, which may then be used to limit its occurrence in susceptible breeds.

The diagnosis of IE in young individuals remains primarily based on owner observations and/or film recordings. The latter are not always available. If it is not available, clinicians can show owners recorded film material of seizuring dogs. This was done in this study, hence the diagnosis, together with the additional physical and neurological examination and blood/urine examination reached a tier 1 confidence level for the diagnosis of IE, or EUC in the older dogs. Although tier II and III confidence levels provide greater accuracy of IE diagnosis, they are cost-intensive, often rendering them impossible.

One of the limitations of this study was the means and time of data collection. Considering that the data was extrapolated from questionnaires filled out subjectively, the reliability of the data provided must be assessed critically. Also, focal seizures or focal seizures preceding a generalized tonic-clonic seizure may have been missed or unrecognized by the owners. The questionnaires were collected at a single time point, at various stages of the dogs' disease process, which may have influenced the answers. Creating clear inclusion criteria was an attempt to objectify this data. Features such as response to treatment, quality of life or survivability were not assessed in this study. A follow-up study at a second time point assessing the long-term outcome of IE in this population of dogs may be useful.

## Conclusion

We reported IE and EUC in a Dutch population of IS based on owner questionnaires. The ES in this breed were predominantly generalized tonic-clonic seizures with post-ictal signs in the majority of the dogs, more commonly occurring in males than females. They also featured a later-onset compared to ES in other reported breeds. A strong genetic component is suspected in the breed, and the high percentage of CS supports the implementation of early ASM. A further study of IE, coupled with a genetic study, needs to be performed on a larger population of IS to identify causative genetic variants.

## Data availability statement

The raw data supporting the conclusions of this article will be made available by the authors, without undue reservation.

## Ethics statement

Ethical review and approval was not required for the animal study because it is a retrospective study performed on cases presented for veterinary care. Written informed consent was obtained from the owners for the participation of their animals in this study.

## Author contributions

PM was responsible for the study conception and data collection in cooperation with MP, MD-E, QS, KS, and PL. The cases were either seen by MP, PM, MD-E, QS, and KS. Statistical analysis, data analysis, and manuscript writing were performed by MP, KS, and PM. PM supervised data analysis and manuscript editing, in cooperation with MD-E, QS, and PL. All authors contributed to the article and approved the submitted version.
