# Corrigendum: Phenotypic characterization of idiopathic epilepsy and epilepsy of unknown cause in Irish Setters

**DOI:** 10.3389/fvets.2023.1145556

**Published:** 2023-03-06

**Authors:** Marta Plonek, Montse M. Diaz-Espineira, Quirine E. M. Stassen, Koen M. Santifort, Peter A. J. Leegwater, Paul J. J. Mandigers

**Affiliations:** ^1^Neurology Section, Evidensia Hospital Arnhem, Arnhem, Netherlands; ^2^Department of Clinical Sciences, Faculty of Veterinary Medicine, Utrecht University, Utrecht, Netherlands

**Keywords:** canine epilepsy, dog, idiopathic epilepsy, epileptic seizure, hereditary

In the published article, there was an error in Figure 2 and its caption as published. Two dams were marked as affected with IE, when they did not in fact have IE and were supposed to only be marked as parents of dogs with IE.

The corrected Figure 2 and its caption appear below.

**Figure 2:**
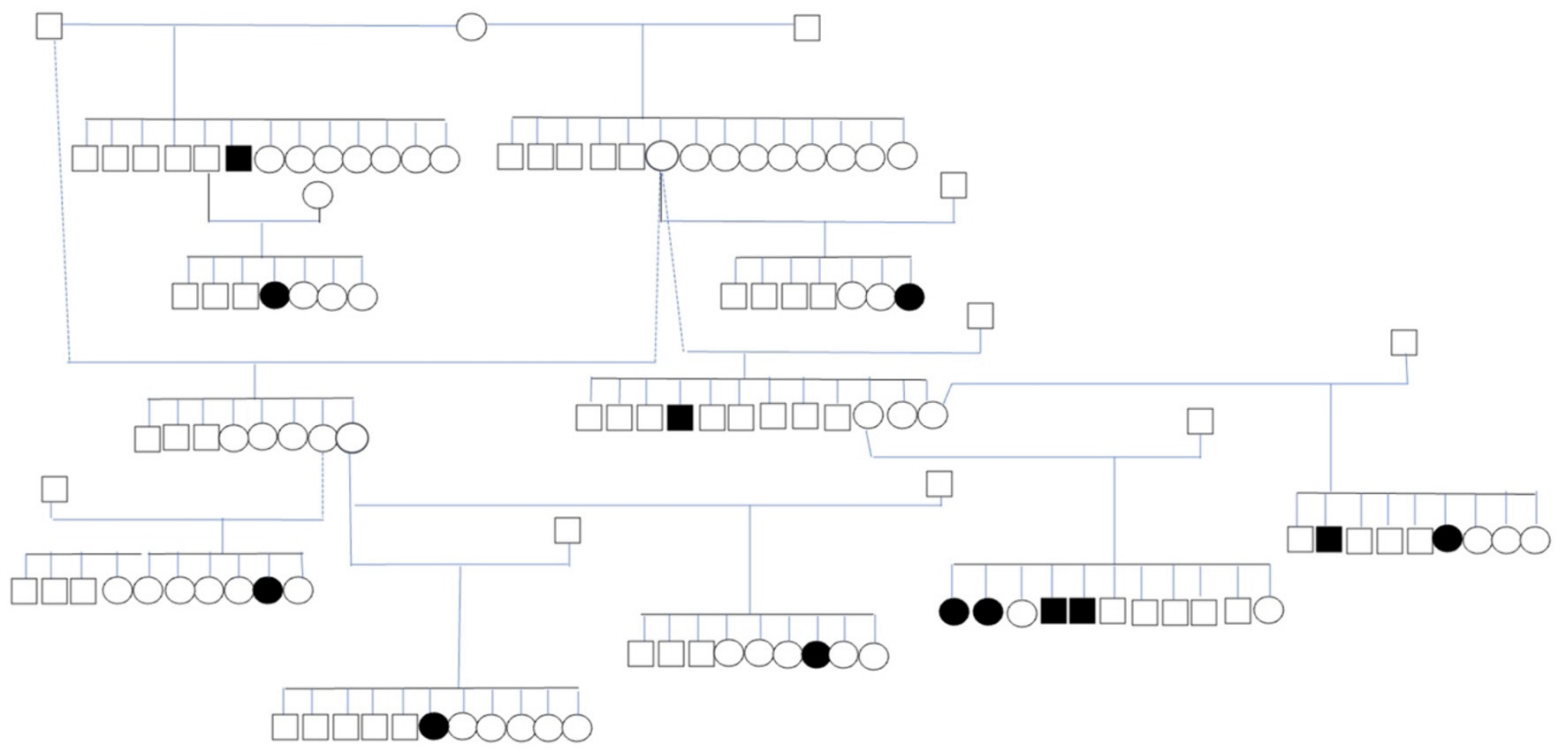
Subpopulation of IS with 13 affected dogs. The circles represent females, the squares represent males, shapes filled in black represent dogs with ES.

In the published article, there was an error in Table 4 as published. The segregation analysis should include 11 litters instead of 10 litters. The corrected Table 4 and its caption appear below.

**Table 4:** Segregation analysis of the studied affected littermates.

**Litter**	**Affected siblings**	**Unaffected siblings**	**% Affected in litter**
1	1	12	7.6%
2	0	14	0%
3	1	6	14.2%
4	1	6	14.2%
5	0	8	0%
6	1	11	8.3%
7	1	9	10%
8	1	10	9.1%
9	1	8	11.1%
10	4	7	36.3%
11	2	7	22.2%
Total affected	13	98	11.7%

In the published article, there was an error in the main text.

A correction has been made to the section Pedigree analysis, line 181. This sentence previously stated:

“Thirteen affected dogs from 10 litters were closely related (Figure 2).”

The corrected sentence appears below:

“Thirteen affected dogs from 11 litters were closely related (Figure 2).”

A correction has been made to the section Pedigree analysis, line 184. This sentence previously stated:

“Two affected bitches produced five litters each with IE dogs, two of these litters were from the same sire.”

The sentence should be deleted.

The authors apologize for this error and state that this does not change the scientific conclusions of the article in any way. The original article has been updated.

